# Structure-based medical acupuncture for trigeminal neuralgia secondary to lateral medullary syndrome: a case report

**DOI:** 10.1186/s13256-026-05957-5

**Published:** 2026-03-24

**Authors:** Kaixuan Ma, Jiawei Shi, Linru Hou, Yuting Wang, Zifeng Dai, Yuzheng Du, Lili Zhang

**Affiliations:** 1 First Teaching Hospital of Tianjin University of Traditional Chinese Medicine, Tianjin, China; 2 National Clinical Research Center for Chinese Medicine, Tianjin, China; 3 Tianjin University of Traditional Chinese Medicine, Tianjin, China

**Keywords:** Structure-based medical acupuncture, Lateral medullary syndrome, Trigeminal neuralgia, Case report

## Abstract

**Introduction:**

Lateral medullary syndrome, a common type of posterior circulation stroke, typically presents with classic symptoms including vertigo, gait ataxia, hoarseness, and dysphagia. In several cases, it may also lead to ipsilateral facial pain consistent with trigeminal neuralgia. Current management mainly involves pharmacological or surgical interventions, which are often associated with adverse effects and limited efficacy.

**Case presentation:**

A 52-year-old ethnic Han male patient with trigeminal neuralgia secondary to lateral medullary syndrome received structure-based medical acupuncture. After a 2-week intervention (six sessions per week), the patient’s numerical rating scale score improved from 8 to 2, with a significant reduction in pain episode frequency. No adverse events were reported during treatment.

**Conclusion:**

Structure-based medical acupuncture, as an adjunct to conventional care, may alleviate facial pain symptoms in patients with trigeminal neuralgia secondary to lateral medullary syndrome. It may represent an effective complementary and alternative therapy option. Further rigorous, controlled clinical trials are warranted to validate its efficacy.

## Introduction

Medullary infarction accounts for approximately 7.0% of all posterior circulation ischemic strokes [1]. Lateral medullary syndrome (LMS), also known as Wallenberg’s syndrome, is the most common type of medullary infarction, comprising about 72.7% of cases [2], with diffusion-weighted imaging (DWI) being the most reliable modality for its detection [3]. The most common symptoms of onset are dizziness with vertigo, loss of balance with gait instability, hoarseness of voice, and difficulty swallowing [4], although clinical presentations can be partial.

Beyond these classic manifestations, LMS can also present with less common symptoms, such as ipsilateral facial pain [4], which may present as secondary trigeminal neuralgia (TN) [5]. Studies show that lesions in the dorsolateral brainstem involving the trigeminal descending tract and the trigeminal spinal nucleus result in ipsilateral facial pain [6, 7]. TN often significantly impairs patients’ quality of life. First-line management typically includes medications such as carbamazepine or gabapentin [8–10], which carry risks of adverse effects [11]. For refractory cases, surgical options exist but lack robust evidence and pose potential complications [11].

Given the limitations of conventional therapies, there is a compelling need to explore alternative treatment modalities. Structure-based medical acupuncture (SMA) is an anatomy-guided approach grounded in modern medical science that has gained increasing clinical adoption in recent years [12]. The therapeutic potential of SMA for LMS secondary TN remains currently underappreciated. In this case, a 52-year-old male with TN secondary to LMS showed a significant reduction in both pain intensity and attack frequency following a treatment course that included SMA. The report follows the CARE reporting checklist.

## Case presentation

**Fig. 1:**
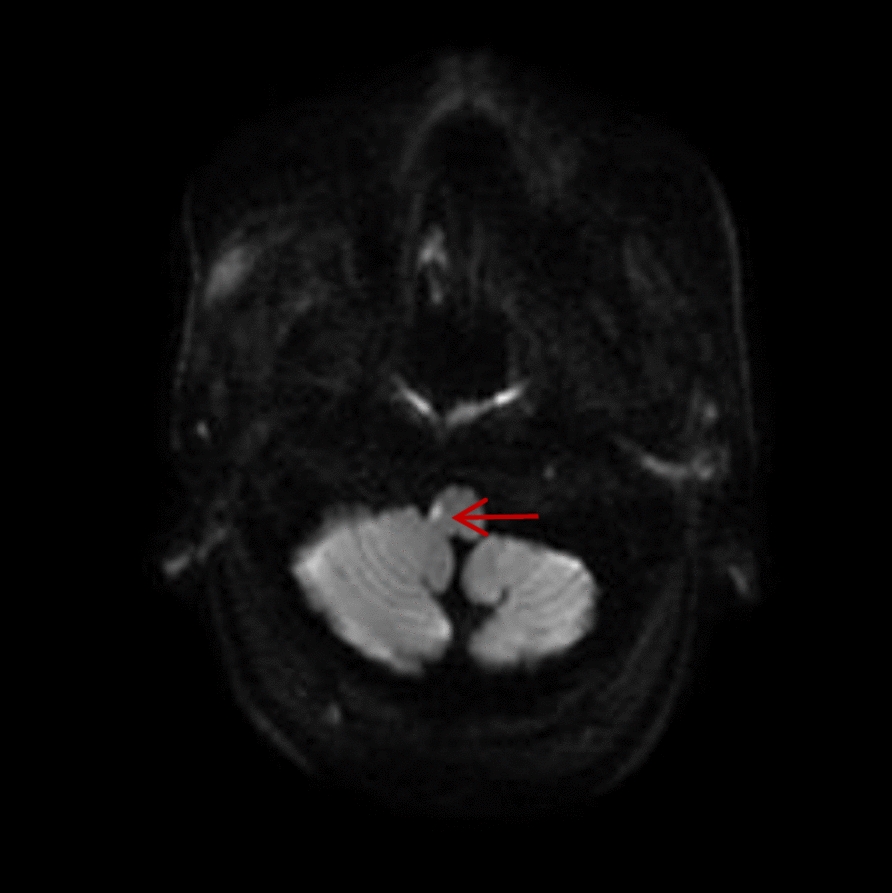
Diffusion-weighted imaging upon diagnosis of lateral medullary infarction. The red arrow indicates the area of high signal intensity in the right lateral medulla

On 22 January 2025, a 52-year-old ethnic Han male patient was admitted to cardiovascular department of the First Affiliated Hospital of Tianjin University of Traditional Chinese Medicine with dizziness and lower limb weakness for 2 days. The patient had a history of poorly controlled hypertension. Brain magnetic resonance imaging demonstrated high signal intensity in the right lateral medulla on DWI (Fig. 1), confirming LMS. After 11 days of inpatient routine treatment, including antiplatelet, lipid regulation, blood pressure control, antidizziness, and improvement of cerebral circulation therapy, the patient was discharged with improvement of the above symptoms.

However, 1 week post discharge, he developed severe paroxysmal, lancinating pain in the right frontal region, occurring three to four times daily for 1–2 min each, along with aggravation of dizziness and irritability. The severity was rated as 8 on the numerical rating scale (NRS). He was hospitalized in the Department of Acupuncture and Moxibustion of the First Affiliated Hospital of Tianjin University of Traditional Chinese Medicine on 10 February 2025. Physical examination confirmed right-sided Horner’s syndrome (ptosis and miosis, diminished temperature sensation in the left lower extremity) and hypertensive status (BP 170/80 mmHg). On the basis of the characteristic pain distribution, quality, and frequency—along with supporting neuroimaging findings—the patient was diagnosed with TN involving the ophthalmic branch (V1), secondary to LMS.

The conventional therapeutic regimen consisted of: intravenous infusion of Xueshuantong (lyophilized) for injection (500 mg once daily) and Danhong injection (40 ml once daily); oral administration of butylphthalide soft capsules (0.2 g three times daily) for circulation improvement; dual antiplatelet therapy with oral clopidogrel sulfate tablets (75 mg once daily) and aspirin enteric-coated tablets (0.1 g once daily); lipid management with oral atorvastatin calcium tablets (20 mg at bedtime); blood pressure control with oral amlodipine besylate tablets (5 mg once daily); and symptomatic relief of vertigo with oral flunarizine hydrochloride capsules (5 mg at bedtime). No analgesic drugs were used during the treatment period.

**Fig. 2:**
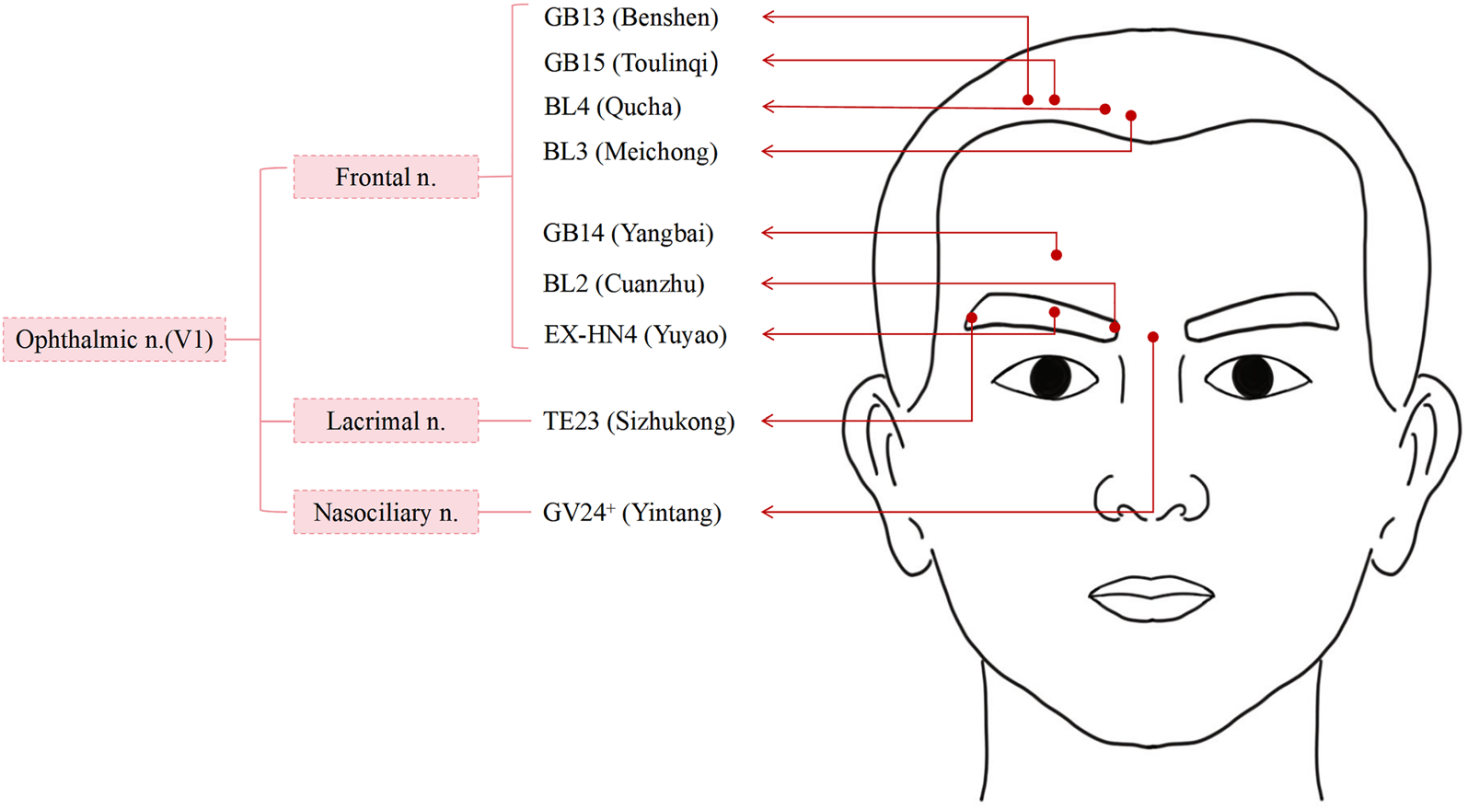
Facial acupoint locations and their nerve innervation

Standard acupuncture for stroke was combined with SMA targeting the trigeminal anatomy: GV20 (Baihui), EX-HN1 (Sishencong), Ex-HN4 (Yuyao), GB14 (Yangbai), GB13 (Benshen), GB15 (Toulinqi), BL2 (Cuanzhu), BL3 (Meichong), BL4 (Qucha), TE23 (Sizhukong), GV24^+^ (Yintang), LU5 (Chize), LI4 (Hegu), ST36 (Zusanli), ST37 (Shangjuxu), ST39 (Xiajuxu), SP9 (Yinlingquan), SP6 (Sanyinjiao), LR3 (Taichong), and GB41 (Zulinqi) (Fig. 2), with 30-min needle retention per session. Sterile disposable needles (0.25 × 40 mm; Huatuo, Suzhou Medical Supplies Factory Co., Ltd., China) were employed. Facial acupoints at 0.3–0.5 cun (approximately 0.8–1.3 cm) and non-facial points at 1.0–1.5 cun (approximately 2.5–3.8 cm). Following needle insertion, standardized manual stimulation was performed using a combination of lifting–thrusting and rotating techniques until achieving deqi sensation, operationally defined as the elicitation of characteristic needling sensations (soreness, numbness, heaviness, or distension) reported by participants. The treatment cycle lasted 2 weeks, with six sessions administered per week.

By day 7, clinical assessment revealed marked pain reduction (NRS 5) with decreased frequency (1–2 episodes/day) and improved vertigo, and a notable alleviation of irritability. At day 14, further symptomatic improvement was documented with pain decreasing to NRS 2 occurring only every 2–3 days, complete resolution of vertigo, and stabilized mood, prompting discharge with substantial clinical improvement. The patient was discharged with substantial improvement. No adverse events from acupuncture were observed. Post discharge, oral medications continued, and outpatient acupuncture was advised at three sessions weekly.

## Discussion

LMS typically results from infarction in the posterior inferior cerebellar artery (PICA) and the vertebral artery [7, 8]. TN secondary to brainstem infarction, though uncommon, is documented [13]. Proposed mechanisms include demyelination-induced abnormal electrical impulses [14], scar tissue hypersensitivity [9], or ephaptic transmission [15]. While no prior studies specifically address acupuncture for LMS-related TN, some evidence supports its use in classical TN [16]. In this case, a 2-week course of SMA, along with conventional therapy, was associated with a reduction in both pain intensity (NRS 8 to 2) and frequency in a patient with LMS-related TN. This occurred without adverse effects, suggesting that SMA may be a useful adjunct in this condition. The core premise of SMA is its anatomical basis, a concept supported by current findings, which demonstrate that acupoints correspond to distinct neuroanatomical structures [17]. This correlation provides precise therapeutic access to conditions involving the trigeminal nerve (CN V) branches in the facial region (Fig. 2). The frontal nerve, lacrimal nerve, and nasociliary nerve are terminal branches of the ophthalmic nerve (V1), with the supraorbital nerve constituting a major branch of the frontal nerve. These innervation patterns are reflected in specific acupoints: Ex-HN 4 is located at the midpoint of the eyebrow, vertically aligned with the pupil during forward gaze, and is associated with the supraorbital nerve. GB 14 lies 1 cun above the eyebrow midpoint, approximately one third of the distance from the eyebrow to the anterior hairline, and is innervated by the common calvarial branch of the supraorbital nerve. GB 13 is situated 0.5 cun within the forehead hairline at two-thirds the distance from GV 24 to ST 8, corresponding to the lateral branch of the supraorbital nerve. GB 15 is positioned directly superior to GB 14, 0.5 cun within the hairline midway between GV 24 and ST 8, and relates to the medial branch of the supraorbital nerve. BL 2 is found at the medial eyebrow terminus or supraorbital notch, innervated by the supratrochlear nerve (a terminal branch of the frontal nerve). BL 3 lies superior to BL 2, positioned 0.5 cun within the anterior hairline between GV 24 and BL 4. BL 4 is located 1.5 cun lateral to the midline at the anterior hairline, one third of the distance from GV 24 to ST 8. Both BL 3 and BL 4 are innervated by the calvarial branch of the supratrochlear nerve. Furthermore, TE 23 at the lateral eyebrow depression connects to the lacrimal nerve, and Ex-HN 3 between the eyebrows at the glabellar midline links to the nasociliary nerve. These precise neuroanatomical correlations underscore the mechanistic basis for acupoint efficacy in managing ophthalmic division (V1)-related pathologies [17].

Acupuncture alleviates TN through multimodal actions—encompassing endogenous opioid activation [18, 19], inflammatory regulation [20], and central neuromodulation [19]—collectively contribute to acupuncture’s analgesic efficacy in TN through neurohumoral mechanisms [21], offering both symptomatic relief and improved quality of life for patients.

This report has several limitations: the observation period was short, with no long-term follow-up or objective outcome measures beyond patient-reported pain scores; and the single-case, uncontrolled design precludes causal inference. It is crucial to note that in such an uncontrolled case report, the observed improvement cannot be definitively attributed to SMA alone, as it may be influenced by the natural history of stroke recovery, concurrent medications, or other factors.

## Conclusion

LMS has a better functional outcome than most other stroke syndromes. Most patients can return to satisfactory activities of daily living [4]. Nevertheless, the associated TN severely compromises both the affective state and quality of life in affected patients, requiring prompt medical intervention. This case illustrates the favorable therapeutic efficacy of SMA, as an adjunct to conventional care, in mitigating TN pain in a patient with LMS. These findings are merely hypothesis-generating. Further investigation through rigorous, controlled clinical trials is necessary to evaluate any potential efficacy.

## Data Availability

All data available are included in this article and its supplementary material files. Further inquiries can be directed to the corresponding author.

## Abbreviations

SMA: Structure-based medical acupuncture
LMS: Lateral medullary syndrome
DWI: Diffusion-weighted imaging
TN: Trigeminal neuralgia
NRS: Numerical rating scale
